# An Integrated Approach to the Design of PHBV-Based Blends: Structure–Property–Performance Relationships for Compostable Packaging

**DOI:** 10.3390/polym18121426

**Published:** 2026-06-07

**Authors:** Karlo Grgurević, Martina Miloloža Nikolić, Dajana Kučić Grgić, Vesna Ocelić Bulatović

**Affiliations:** Faculty of Chemical Engineering and Technology, University of Zagreb, Trg Marka Marulića 19, 10000 Zagreb, Croatia; kgrgurevi@fkit.unizg.hr (K.G.); miloloza@fkit.unizg.hr (M.M.N.); dkucic@fkit.unizg.hr (D.K.G.)

**Keywords:** poly(3-hydroxybutyrate-co-3-hydroxyvalerate), polylactide, polymer blends, biodegradable polymers, compostable packaging

## Abstract

Environmental concerns with petroleum-based polymers have accelerated the development of biodegradable alternatives, making poly(3-hydroxybutyrate-co-3-hydroxyvalerate) (PHBV) a promising candidate for sustainable packaging. However, its functional performance necessitates modification through blending. In this study, blends containing 65–85 wt.% polylactide (PLA) were investigated to establish structure–property relationships relevant to compostable packaging. The results reveal partial miscibility of the blends and pronouncedcomposition-dependent changes in morphology and thermal behavior, characterized by an increase in glass transition temperature and a decrease in PLA melting temperature. Increasing PLA content (≥80 wt.%) enhanced thermal stability, increasing the degradation temperature to 288.0 °C. In contrast, higher PHBV content (≥25 wt.%) significantly improved barrier properties of PLA, reducing oxygen and water vapor transmission rates to 74.47 cm^3^ m^−2^ day^−1^ and 29.11 g m^−2^ day^−1^, respectively. Biodegradation behavior revealed complete degradation of PHBV after 56 days, whereas PLA showed only 1.29% mass loss under identical conditions. In the blends, biodegradation proceeded preferentially through the PHBV phase, resulting in composition-dependent mass loss. Among the investigated compositions, PLA65/PHBV provided the most balanced combination of barrier performance, mechanical behavior, and biodegradation response. Overall, these findings demonstrate that tailoring composition enables the design of polymer systems for sustainable packaging applications.

## 1. Introduction

Due to the continuously increasing global demand for food packaging materials, the packaging industry has become one of the largest and most commercially significant industrial sectors worldwide. In 2020, approximately 2.11 million tons of plastic materials were produced, with food packaging accounting for nearly 47% of total plastic consumption. Most packaging materials are still derived from petroleum-based polymers because of their favorable mechanical and barrier properties, cost-effectiveness, and broad availability [1]. However, long-term persistence in the environment and accumulation in terrestrial and marine ecosystems represent major environmental concerns with serious implications for biodiversity and human health [2]. Consequently, biodegradable and bio-based polymers, particularly polylactide (PLA) and polyhydroxyalkanoates (PHAs), have attracted considerable attention as sustainable alternatives for next-generation packaging materials [3,4]. In addition to biodegradability and biocompatibility, such materials may also provide antioxidant and antimicrobial functionalities, further increasing relevance for food packaging applications [5].

Polyhydroxyalkanoates are biopolymers synthesized by microorganisms as intracellular energy storage compounds [6]. Depending on the carbon chain length of their monomeric units, PHAs are classified into short-, medium-, and long-chain-length polymers [7]. Among short-chain-length PHAs, poly(3-hydroxybutyrate-co-3-hydroxyvalerate) (PHBV) is one of the most extensively studied materials for packaging applications. PHBV is a copolymer composed of hydroxybutyrate and hydroxyvalerate characterized by high crystallinity and a glass transition temperature ranging from −5 °C to 20 °C, depending on hydroxyvalerate content [8,9]. Compared to poly(hydroxybutyrate) (PHB), PHBV exhibits lower brittleness and improved toughness. Increasing hydroxyvalerate content reduces crystallinity, melting temperature, and tensile strength while enhancing flexibility and biodegradation rate [10]. Under aerobic conditions, PHBV undergoes relatively rapid biodegradation through the enzymatic action of microbial depolymerases capable of hydrolyzing ester bonds into water-soluble degradation products [8].

PLA is another widely investigated biodegradable polymer frequently used in food packaging applications. PLA belongs to the family of aliphatic polyesters synthesized from lactic acid obtained either by carbohydrate fermentation or chemical synthesis [11,12]. Processing can be performed in both amorphous and semicrystalline forms, with crystallinity levels reaching approximately 40%, while crystallization may occur in different polymorphic structures (α, β, and γ), of which the α-form is thermodynamically the most stable [13]. Crystalline regions contribute to improved thermal stability and mechanical strength but simultaneously reduce biodegradation rates [14]. Despite numerous advantages, both PLA and PHBV still suffer from several inherent limitations, including brittleness, limited thermal resistance, and inadequate gas barrier performance, which restricts their broader implementation in food packaging applications [5]. Such limitations become particularly critical in packaging systems requiring controlled gas permeability, mechanical durability, and prolonged shelf life.

One of the most effective strategies for overcoming these drawbacks is polymer blending. By combining two polymers, material properties can be tailored to achieve synergistic performance. However, blend behavior strongly depends on the miscibility and interfacial interactions of the components. Miscible blends exhibit a single glass transition temperature (*T*_g_), whereas immiscible systems undergo phase separation and display multiple *T*_g_ values. Partially miscible systems show intermediate behavior, often leading to complex structure–property relationships [15,16,17].

PLA/PHBV blends have attracted increasing attention as promising candidates for sustainable packaging applications due to relatively high stiffness and thermal stability of PLA, and enhanced biodegradability and improved gas barrier properties of PHBV. Nevertheless, most previously reported studies primarily examine individual properties, such as thermal behavior, mechanical response, barrier performance, or biodegradation separately. Correlations between blend composition, phase structure, crystallization behavior, functional performance, and end-of-life degradation remain comparatively less explored, despite being essential for the rational design of compostable packaging materials. Composition-dependent phase morphology plays a particularly important role in determining gas transport behavior, mechanical integrity, and biodegradation kinetics of PLA/PHBV systems. Partial miscibility between PLA and PHBV may simultaneously influence crystallinity development, interfacial adhesion, and degradation pathways, leading to complex performance trade-offs highly relevant for packaging applications. Understanding such interrelationships is especially important in biodegradable packaging systems, where functional performance during use must be balanced with efficient degradation after disposal. Although PHBV is generally recognized as highly biodegradable, complete degradation under controlled composting conditions remains strongly dependent on the biological activity of the degradation medium. Biologically enriched compost systems therefore provide valuable insight into degradation mechanisms and degradation efficiency relevant for realistic end-of-life scenarios of compostable packaging materials. Accordingly, PLA/PHBV blends containing different PHBV fractions were investigated through an integrated evaluation of structural evolution, thermal behavior, mechanical response, barrier performance, and biodegradation behavior in order to establish comprehensive structure–property–performance relationships relevant to compostable food packaging applications. Particular attention was directed toward understanding how phase morphology, crystallization behavior, and partial miscibility govern the balance between functional performance during use and degradation behavior under composting conditions.

## 2. Materials and Methods

### 2.1. Materials

Polymer blends were prepared with Luminy^®^ LX175 PLA, purchased from the TotalEnergies Corbion Ltd. (Rayong, Thailand), supplied in the form of transparent granules. PHBV (ENMAT™ Y1000P) was purchased from the TianAn Biologic Materials Co., Ltd. (Ningbo, China) and was supplied as yellow pellets. The general properties of PLA and PHBV are presented in Table 1.

### Preparation of PLA/PHBV Polymer Blends

PLA/PHBV polymer blends with different compositions (65/35, 70/30, 75/25, 80/20, and 85/15) were prepared by melt blending neat PLA and PHBV in a Brabender kneader at 180 °C and 60 rpm. Neat PLA and PHBV were also processed under identical conditions. The prepared blends were denoted as PLAX/PHBV, where X represents the PLA content (65–85 wt.%). To obtain thin films, manually shredded blend granules were compression-molded between two Teflon foils places on metal using a Fontune laboratory hydraulic press, model SRB 140 (EC 320 × 320 NB) (Rotterdam, The Netherlands), at 180 °C and 25 kPa for 3 min, including a preheating period of 1 min. For mechanical testing, the blend granules were compression-molded in a mold between Teflon foils for 5 min with a 1 min preheating. All samples were subsequently cooled under pressure using on a Dake Model 44-226 hydraulic press (Dake Corporation, Grand Haven, MI, USA).

### 2.2. Characterization Methods

#### 2.2.1. Scanning Electron Microscopy (SEM)

Sample morphology was analyzed using a scanning electron microscope (SEM Tescan Vega III, Brno, Czech Republic) operated at an accelerating voltage of 20 kV. Brittle fracture surfaces were prepared through cryogenic fracturing after immersion of the samples in liquid nitrogen. Prior to the SEM analysis, the fractured surfaces were mounted on aluminum stubs and sputter-coated with a thin layer of gold and platinum to ensure adequate electrical conductivity. Surface morphology was examined at a magnification of 500×.

#### 2.2.2. Attenuated Total Reflectance–Fourier Transform Infrared Spectroscopy (ATR-FTIR)

Attenuated Total Reflectance–Fourier Transform Infrared Spectroscopy (ATR-FTIR) was employed to evaluate changes in chemical structure and to obtain information regarding the chemical composition of the polymer blends and neat samples. Spectra were recorded using a PerkinElmer Spectrum Two spectrophotometer (Waltham, MA, USA) equipped with an ATR accessory. Measurements were performed at room temperature in the wavenumber range of 4000 to 650 cm^−1^ with a spectral resolution of 4 cm^−1^ and 32 scans per measurement. To monitor chemical changes occurring during biodegradation, ATR-FTIR spectra were additionally recorded in the range of 4000 to 400 cm^−1^ at a resolution of 4 cm^−1^ using 32 scans per measurement. For this purpose, an IRTraces-100 Shimadzu spectrophotometer (Tokyo, Japan) was used.

#### 2.2.3. Differential Scanning Calorimetry (DSC)

Thermal properties were analyzed through differential scanning calorimetry (DSC) using a Mettler Toledo DSC 3+ (Greifensee, Switzerland). Measurements were performed in the temperature range from −50 to 200 °C at a heating rate of 10 °C min^−1^, including isothermal holding periods of 3 min at both −50 and 200 °C. Double heating and cooling cycles were applied, while characteristic thermal parameters were determined from the second heating cycle. All measurements were conducted under a nitrogen atmosphere with a gas flow rate of 60 mL min^−1^. The degree of crystallinity (*χ*_c_) of the samples was calculated from obtained melting enthalpy values according to Equation (1):

(1) $$\chi_{c}/\%\;=\frac{\Delta H_{m}}{\Delta H_{m}^{{}^{\circ}}\times w(polymer)}\times 100\%$$

where *w* represents the mass fraction of PLA or PHBV, Δ*H_m_*/J g^−1^ the melting enthalpy of the samples, and $\Delta H_{m}^{{}^{\circ}}/J\;g^{-1}$ represents the melting enthalpy of a 100% crystalline polymer. According to the literature, the values of 93.1 J g^−1^ and 109 J g^−1^ were used for PLA and PHBV, respectively [16,17]. DSC curve were used to determine crystallization and melting behavior of the investigated systems. Crystallization and melting temperatures were identified from maxima of the corresponding thermal transitions, while the areas under the peaks were used to calculate the enthalpies of crystallization (∆*H_c_*) and melting (Δ*H_m_*).

#### 2.2.4. Thermogravimetric Analysis (TGA)

The thermogravimetric analysis was performed using a Mettler Toledo TGA/DSC 3+ instrument (Greifensee, Switzerland) in order to evaluate the thermal stability of the investigated samples. Measurements were carried out in the temperature range from 25 to 700 °C at a heating rate of 10 °C min^−1^ under a nitrogen atmosphere with a flow rate of 60 mL min^−1^.

#### 2.2.5. Dynamic Mechanical Analysis (DMA)

Dynamic mechanical analysis was conducted to evaluate the viscoelastic behavior of neat polymers and PLA/PHBV blends. Temperature sweep measurements were performed in tensile mode at a frequency of 1 Hz and an amplitude of 5 μm. The samples were heated from −30 to 150 °C at a heating rate of 3 °C min^−1^. The dimensions of the samples’ cross-sections were approximately 1 mm × 10 mm, while the gauge length was 20 mm. Storage modulus, (*E*′), loss modulus (*E*″), and damping factor (tan δ) were determined using a Discovery HR30 Hybrid Rheometer (TA Instruments, New Castle, DE, USA).

#### 2.2.6. Tensile Test

Tensile properties of PHBV-based blend films were determined using a universal testing machine (Zwick 147670 Z100/SN5A, Ulm, Germany) equipped with a 2000 N load cell. Measurements were carried out at 23 °C and 65% relative humidity in accordance with ISO 527-1:2019 [18]. The tests were performed at a crosshead speed of 50 mm min^−1^. Film thickness was measured at three different locations on each specimen, and the average value was used for calculations. At least five specimens per formulation were tested to ensure reproducibility, and the reported values represent mean results with standard deviations below 5%.

#### 2.2.7. Gas Permeability Testing

Barrier properties of the prepared blends were evaluated through water vapor transmission rate (WVTR) and oxygen transmission rate (OTR) measurements. Thin films of the prepared polymer blends (thickness approximately 100 μm; area approximately 50 cm^2^) were analyzed using a C403H OTR/WVTR Testing System (Labthink Instruments Co., Ltd., Jinan, China). WTVR measurements were taken according to ASTM F1249 standard [19] at 38.0 °C and 90.0% relative humidity, where OTR measurements were performed according ASTM D3985 standard [20] at 23.0 °C and 0.0% relative humidity. All measurements were carried out in triplicate to ensure reproducibility.

#### 2.2.8. Biodegradation in Soil

To evaluate the biodegradable behavior of neat polymers and their blends, samples with dimensions of 15 mm × 15 mm × 0.1 mm were prepared from each investigated material. The samples were buried in garden soil at a depth of 10 cm for 56 days in accordance with ISO 17556:2019 [21]. Biodegradation experiments were conducted using commercial compost soil (ZRINKO KOMPOST, Zagrebački holding, Zrinjevac, Croatia), selected as an environmentally relevant medium for assessing degradation behavior of the investigated materials. The compost soil exhibited a pH value of 6.52 and a moisture content of 55.34%, providing favorable conditions for microbial growth and biodegradation activity. To ensure the compost soil was additionally inoculated with selected microorganisms known for their biodegradation potential. The bacterial strains included *Bacillus subtilis* and *Pseudomonas aeruginosa*, while the fungal cultures comprised *Aspergillus niger* and *Trichoderma viride*. The initial concentration of each inoculated microbial culture was 10^6^ CFU (g dry matter)^−1^. The selection of the microorganisms was based on their ability to produce extracellular enzymes involved in the degradation of polymeric and organic materials. In addition to the inoculated cultures, the indigenous microbial population naturally present in the compost soil was determined prior to the experiments. The initial bacterial count was 2.56 × 10^7^ CFU (g dry matter)^−1^, whereas the fungal population reached 1.56 × 10^6^ CFU (g dry matter)^−1^. The combination of indigenous microbiota and inoculated bacterial and fungal cultures enable the formation of a diverse microbial consortium capable of enhancing biodegradation efficiency.

The samples were periodically removed after 7, 14, 21, 42, and 56 days, cleaned, dried, and weighed to determine mass loss during biodegradation. The degree of degradation was quantified according to Equation (2):

(2) $$∆m=\;\frac{\left(m_{0}-\;m_{t}\right)}{m_{0}}\times 100\%$$

where *m*_0_ is the initial mass of the sample, while *m_t_* is the mass of the sample after biodegradation.

After removal, all samples were further analyzed using optical (Olympus BX50 light microscope (100×)) (Tokyo, Japan) and polarizing microscopes (Olympus BX53M, 50×, Tokyo, Japan), in order to identify surface damage. Structural changes due to degradation were examined through ATR-FTIR spectroscopy (IRTracer-100, Shimadzu, Japan) as described in Section 2.2.2.

## 3. Results

### 3.1. Scanning Electron Microscopy (SEM)

**Figure 1:**
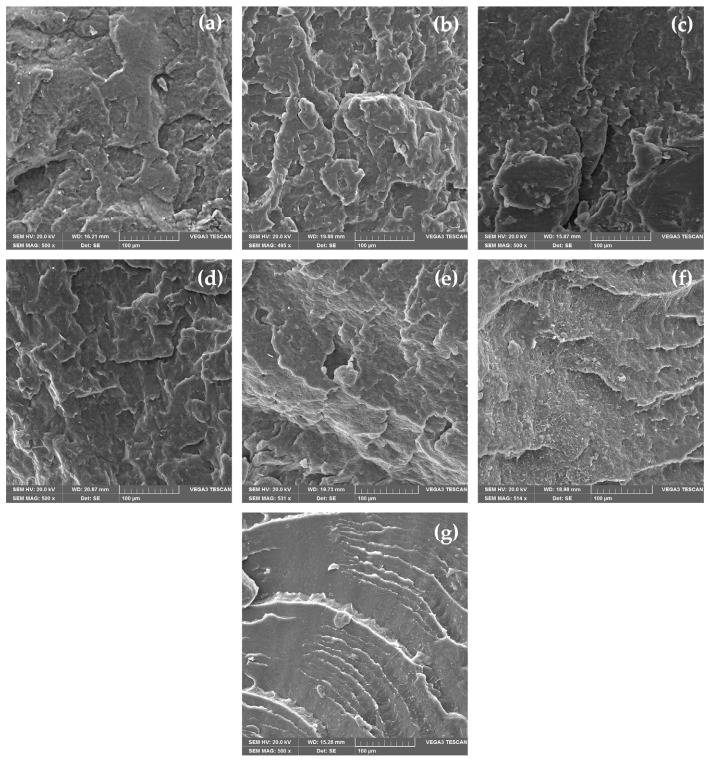
SEM micrographs of the samples: (**a**) PHBV, (**b**) PLA85/PHBV, (**c**) PLA65/PHBV, (**d**) PLA70/PHBV, (**e**) PLA75/PHBV, (**f**) PLA80/PHBV, and (**g**) PLA (all micrographs were recorded at 500× magnification, with a scale bar corresponding to 100 μm).

Figure 1 presents SEM micrographs of the fracture surface for neat PHBV, neat PLA, and PLA/PHBV blends with different blend compositions.

The observed structural homogeneity may be associated with partial interfacial adhesion between PLA and PHBV, arising from the presence of polar carbonyl groups in both polyesters. Such morphology further supports the assumption of at least partial miscibility between the two polymers, consistent with previously reported observations [22]. Zhang and Thomas (2011) [23] reported that PHBV domains can be effectively dispersed within the PLA matrix, contributing to improved structural compatibility and enhanced blend performance.

### 3.2. Attenuated Total Reflectance–Fourier Transformation Infrared Spectroscopy (ATR-FTIR)

**Figure 2:**
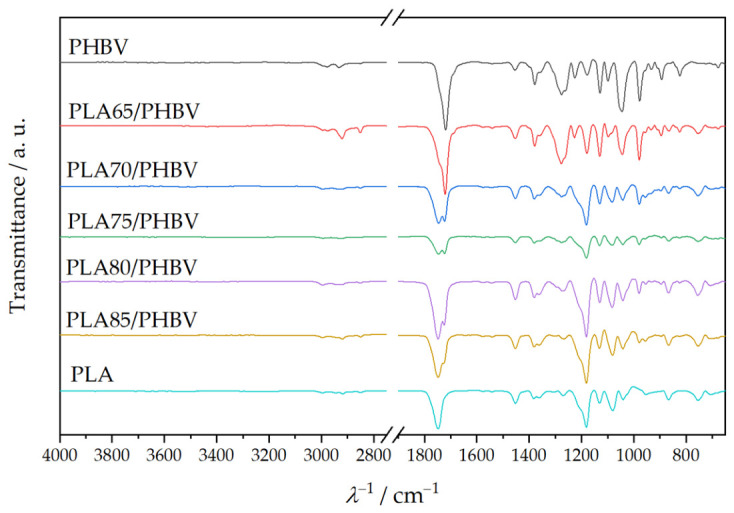
FTIR-ATR spectrum of the tested samples in the wavenumber range of 1800 cm^−1^ to 750 cm^−1^.

Figure 2 shows the ATR-FTIR spectra of the neat PLA, neat PHBV, and the prepared PLA/PHBV blends. In the spectrum of neat PLA, the absorption bands at 2996 cm^−1^ and 2920 cm^−1^ correspond to symmetric stretching vibrations of C–H bonds in CH_3_ and CH_2_ groups, whereas the bands at 2849 cm^−1^, 1452 cm^−1^, and 1357 cm^−1^ are attributed to asymmetric stretching vibrations of CH_3_ and CH_2_ groups. The characteristic absorption band at 1747 cm^−1^ corresponds to stretching vibrations of the ester carbonyl group (C=O). Symetric stretching of C–O–C bonds was detected at 1180 cm^−1^, while asymmetric stretching appeared at 1080 cm^−1^. Additional characteristic bands at 867 cm^−1^ and 754 cm^−1^ are associated with CH_2_ group vibrations [24].

For neat PHBV, the absorption bands at 2976 cm^−1^ and 2933 cm^−1^ correspond to asymmetric stretching vibrations of CH_3_ and CH_2_ groups. The bands at 1452 cm^−1^, 1379 cm^−1^, and 1357 cm^−1^ are attributed to asymmetric bending and symmetric stretching vibrations of C–H bonds within CH_3_ and CH_2_ groups. The intense absorption band at 1719 cm^−1^ corresponds to stretching vibrations of the C=O group. Bands observed at 1275 cm^−1^, 1260 cm^−1^, and 1225 cm^−1^ are associated with symmetric and asymmetric stretching vibrations of C–O and C–O–C groups. The absorption band at 1099 cm^−1^ originates from CH_2_-O-CH_2_ vibrations caused by interactions between C–O and C–C group, while the bands at 1054 cm^−1^ and 1043 cm^−1^ correspond to C–O group stretching vibrations [25].

ATR-FTIR spectra of the blends contain characteristic absorption bands of both neat polymers, while the absence of additional peaks indicates that no chemical reactions occurred during melt blending of PLA and PHBV. Slight shifts in the characteristic carbonyl absorption bands were observed, with the PHBV carbonyl peak shifting from 1719 cm^−1^ to 1722 cm^−1^ and the PLA carbonyl peak from 1747 cm^−1^ to 1750 cm^−1^. Such spectral shifts indicate changes in the local molecular environment associated with crystallization and intermolecular interactions between blend components [23]. A gradual shift in the characteristic bands toward higher wavenumbers with increasing PLA content further suggests the presence of intermolecular interactions between PLA and PHBV. Zhang et al. (2011) [23] proposed that such interactions originate from weak molecular forces between the α-methylene groups of PHBV and the carboxyl groups of PLA. Among the investigated compositions, the PLA75/PHBV blend exhibited the lowest overall band intensity, which may indicate improved blend homogeneity [23]. Furthermore, increased intensity of the absorption bands associated with the amorphous phase at 867 cm^−1^ (C–O–C) and 754 cm^−1^ (C–H) was observed in the blends compared with neat PLA, indicating that incorporation into PHBV disrupts helical crystalline structure of PHBV, promoting the formation of amorphous regions and consequently reducing overall crystallinity [23].

### 3.3. Differential Scanning Calorimetry (DSC)

Differential scanning calorimetry (DSC) was used to evaluate the thermal behavior of neat PLA, neat PHBV. and PLA/PHBV blends. Characteristic thermal parameters, including melting temperature, *T*_m_/°C, glass transition temperature (*T*_g_/°C), crystallization temperature (*T*_c_/°C), cold crystallization temperature (*T*_cc_/°C), melting enthalpies (Δ*H*_m_/J g^−1^), crystallization enthalpies (Δ*H*_c_/J g^−1^), and cold crystallization enthalpy (Δ*H*_cc_/J g^−1^) were determined from the second heating and cooling cycles.

**Figure 3:**
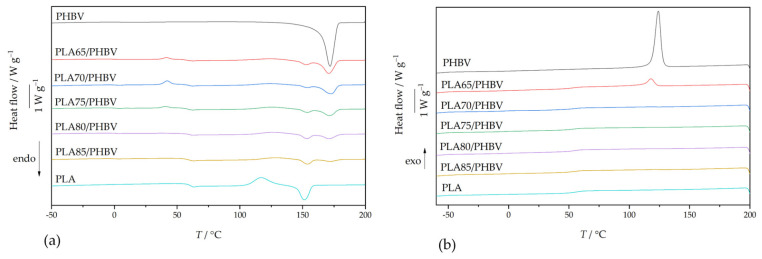
DSC curves of the tested samples: (**a**) heating cycle; (**b**) cooling cycle.

Figure 3a,b present DSC curves of neat polymers and PLA/PHBV blends, while Table 2 summarizes their characteristic phase transitions temperatures, enthalpy, and calculated degrees of crystallinity.

The DSC heating curve of neat PLA shows a glass transition temperature at 59.5 °C, followed by cold crystallization at 116.9 °C. Cold crystallization occurs during heating as increased chain mobility enables molecular rearrangement into a crystalline helical structure. Melting of the formed PLA crystals occurs at 151.4 °C and appears as a single endothermic peak. PLA commonly exhibits double melting behavior associated with the presence of different crystalline modifications (δ and α crystal forms). During heating, less ordered δ crystals melt and recrystallize into more stable α crystals, followed by melting of the α phase. The peak in Figure 3a (light-blue curve) appears as a single peak due to the overlap of the δ and α melting transitions [25]. No crystallization peak was observed during cooling of neat PLA, indicating limited crystallization ability under the applied cooling conditions due to prior cold crystallization during heating. The DSC curve of neat PHBV exhibits a melting peak at 171.9 °C, while the cooling curve shows a crystallization peak at 125.1 °C. The *T*_g_ of PHBV was not clearly detected within the investigated temperature range and therefore was not determined.

In general, the *T*_g_ of polymer blends provides important information regarding polymer miscibility and chain mobility within the amorphous phase [15]. PLA/PHBV blends exhibit two glass transition regions, indicating partial miscibility between the two polymers. The higher *T*_g_ corresponds to the PLA-rich phase, whereas the lower *T*_g_ is associated with the PHBV-rich phase, consistent with literature reports indicating *T*_g_ values of PHBV between −5 and 5 °C [14]. Slight variations in *T*_g_ values with changing blend composition further support the existence of intermolecular interaction between PLA and PHBV [26]. Crystallization behavior strongly depends on blend composition. During cooling, a crystallization peak was observed only for the PLA65/PHBV blend at 118.1 °C corresponding to crystallization of the PHBV phase, considering that neat PLA did not crystallize during cooling and neat PHBV exhibited crystallization at 125.1 °C. In blends containing 70, 75, and 80 wt.% PLA, cold crystallization peaks corresponding to both PLA and PHBV were observed during heating. In contrast, PLA65/PHBV and PLA85/PHBV exhibited only one dominant cold crystallization transition associated with PHBV and PLA, respectively. The thermal transition observed around 40 °C corresponds to cold crystallization of PHBV, whereas the peak around 120 °C is associated with cold crystallization of PLA. Such behavior indicates that incorporation of PLA influences crystallization behavior of PHBV and promotes earlier crystallization within the blends. Heating curves of the blends are characterized by two endothermic melting transitions corresponding to PLA and PHBV phases. The *T*_m_ values of the blends remain relatively close to those of the neat components; however, a slight increase in *T*_m,PLA_ suggests that PHBV may promote crystal growth and ordering within the PLA phase. Even minor shifts in *T*_m_ indicate the presence of intermolecular interactions between the blend components. Generally, the lower values of *T*_m_ correspond to PLA, whereas the higher values originate from PHBV. Polymers with higher melting temperatures crystallize first, forming spherulitic structures that spatially constrain crystallization of the second polymer phase. As a result, the second melting transition becomes more pronounced in the DSC curves. In systems with similar crystallization behavior, partial co-crystallization may occur. Co-crystallization refers to the formation of a crystalline structure containing two different components within the same crystal lattice. Such behavior suggests that PHBV acts as a heterogeneous nucleating agent, promoting crystallization and improving crystal organization of PLA within the blends, particularly at PHBV contents above 20 wt.% [14,26].

The degree of crystallinity strongly depends on polymer chain flexibility and molecular organization and is directly related to melting enthalpy. Both neat PLA and neat PHBV exhibit semicrystalline behavior; however, neat PHBV shows a significantly higher degree of crystallinity. The calculated crystallinity degree of neat PLA (26.4%) is considerably lower than that of neat PHBV (89.5%), indicating a substantially larger amorphous fraction in PLA. Increasing PLA content in the blends leads to lower melting enthalpy values and reduced crystallinity of PHBV due to the increasing contribution of the amorphous PLA phase. Such behavior indicates that the presence of PLA partially restricts growth and organization of PHBV crystalline domains within the blends [14].

### 3.4. Thermogravimetric Analysis (TGA)

Thermal stability of neat PLA, neat PHBV, and PLA/PHBV blends was evaluated by thermogravimetric analysis (TGA). Thermogravimetric (TG) and differential thermogravimetric (DTG) curves were obtained, allowing determination of mass loss during decomposition stages (Δ*m*/%), residual mass at 700 °C (*R*_700°C_/%), onset decomposition temperature (*T*_onset_/°C), temperature of the end of decomposition (*T*_end_/°C), and temperature corresponding to the maximum decomposition rate (*T*_max_/°C).

**Figure 4:**
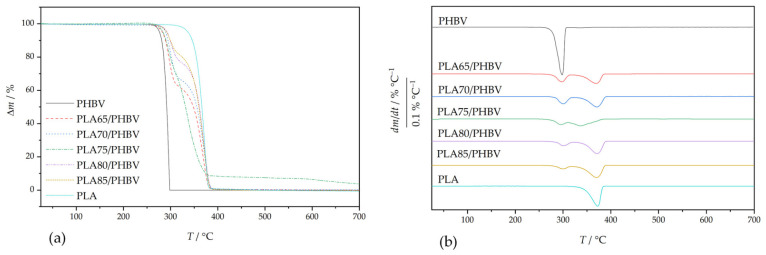
(**a**) TG curves; (**b**) DTG curves of pure PLA and PHBV samples and PLA/PHBV polymer blends.

Figure 4 presents TG and DTG curves of neat polymers and PLA/PHBV blends recorded under a nitrogen atmosphere at a heating rate of 10 °C min^−1^ from room temperature to 700 °C, while the corresponding thermal degradation parameters are summarized in Table 3.

Thermal decomposition of neat PLA proceeds in one step between 340 and 380 °C. The onset decomposition temperature of PLA was determined at 348.5 °C, whereas the degradation process ended at 390.33 °C. The maximum degradation rate occurred at 370.4 °C, corresponding to chain scission reactions and degradation of PLA molecular chains. Neat PLA degraded almost completely (99.2%), leaving a residual mass of 0.4%. Thermal degradation of PLA proceeds predominantly through radical induced degradation mechanism involving ester exchange reactions, resulting in the formation of acetaldehyde, cyclic oligomers, lactide, and carbon monoxide [23]. According to McNeill and Leiper (1985) [27], the degradation of PLA occurs through reverse ester exchange reactions accompanied by cleavage of molecular chains and progressive mass loss [23,27].

Neat PHBV also degraded in a single degradation step but within a considerably narrower temperature range. The onset decomposition temperature of PHBV was 282.4 °C, while the degradation process ended at 303.0 °C. The maximum degradation rate was observed at 298.0 °C. Similar to PLA, PHBV degraded almost completely (98.7%), leaving a residual mass of 1.3%. Thermal degradation of PHBV proceeds through random chain scission at ester groups accompanied by β-hydrogen elimination reactions, resulting in the formation of substituted olefins, crotonic acid, and oligomeric degradation products [10,27].

Thermal degradation of PLA/PHBV blends occurred in two distinct degradation stages. The first degradation stage at lower temperatures corresponds to decomposition of the PHBV phase, whereas the second stage at higher temperatures is associated with the degradation of the PLA phase, consistent with previously reported observations [28]. TG and DTG curves of the blends are positioned between those of neat PLA and neat PHBV, reflecting the contribution of both polymer phases to the thermal degradation behavior. Generally, higher onset degradation temperatures indicate improved thermal stability. Accordingly, neat PLA exhibits significantly higher thermal stability than neat PHBV, primarily due to differences in molecular structure and degradation mechanisms. Among the investigated blends, PLA80/PHBV and PLA85/PHBV exhibited the highest onset and endset degradation temperatures, with PLA80/PHBV showing the highest overall thermal stability. In contrast, PLA70/PHBV exhibited the lowest decomposition temperatures, indicating the lowest thermal stability among the investigated compositions. Increasing PLA content generally contributed to improved thermal stability of the blends, while higher PHBV fractions promoted earlier thermal degradation. Simultaneously, gradual reductions in *T_max_* values associated with the PLA phase indicate that the incorporation of PHBV slightly decreases thermal stability of PLA within the blends. Among all investigated compositions, the PLA75/PHBV blend exhibited the least pronounced DTG peaks, suggesting improved phase compatibility and more homogeneous degradation behavior during thermal decomposition. Such behavior may indicate enhanced intermolecular interactions between PLA and PHBV during melt processing.

### 3.5. Dynamic Mechanical Analysis (DMA)

Dynamic mechanical analysis (DMA) is commonly used to evaluate the viscoelastic behavior of polymeric materials, including neat polymers, polymer blends, and composites. During DMA mesurements, an oscillating force is applied to the sample, and the resulting deformation response is recorded. The viscoelastic behavior of the material is described through the storage modulus (*E*′), loss modulus (*E*″), damping factor (tan δ), and glass transition temperature (*T*_g_). *E*′ represent the elastic energy storing capability of the material, while the *E*″ describes energy dissipation associated with viscous component of the viscoelastic response. Similarly, the damping factor provides information about energy dissipation within the material, where higher tan δ values indicate greater viscous behavior. The position and shape of the *T_g_* peak provide valuable insight into polymer miscibility, molecular mobility, and blend homogeneity [29].

**Figure 5:**
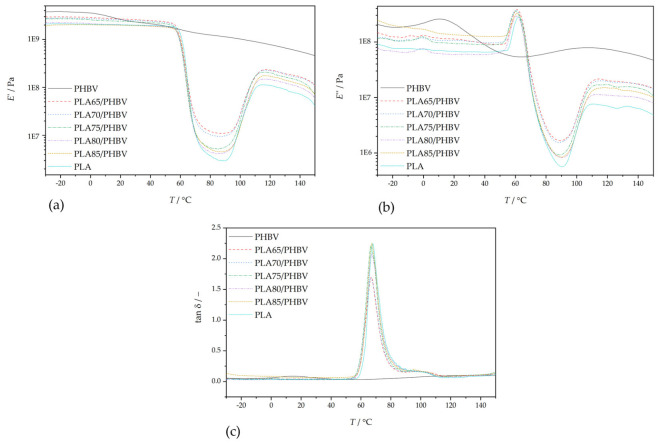
(**a**) *E*′ curves; (**b**) *E*″ curves; (**c**) tan δ curves for the pure PLA and PHBV and their blends.

DMA was performed on the neat PLA, neat PHBV, and PLA/PHBV blends in order to evaluate intermolecular interactions and viscoelastic behavior within the investigated systems. Figure 5a–c present the obtained *E*′, *E*″, and tan δ curves, respectively. Table 4 summarizes the corresponding *T*_g_ values determined from the *E*″ and tan δ curves. As can be seen from *E*″ and tan δ curves of neat PHBV exhibit glass transition temperatures at approximately 14.3 °C and 10.4 °C, respectively. An increase in tan δ curve above approximately 120 °C indicates the occurrence of secondary polymer relaxation associated with crystal–crystal slip. Such behavior refers to the movement of crystalline planes within the crystal structure under applied stress [30]. Additionally, the *E*′ curve shows a continuous decrease after the *T*_g_, corresponding to increased molecular mobility and polymer chain flow with increasing temperature [31]. On the other hand, the *E*′ curve obtained for neat PLA exhibits the characteristic behavior previously described by Cristea et al. [32]. High *E*′ values (approximately 2.1 × 10^9^ Pa) in the low-temperature region correspond to the glassy state, characterized by rigid and brittle polymer behavior. Upon reaching the glass transition region, two *T*_g_ values were determined from *E*″ and tan δ curves at 62.4 °C and 68.0 °C, respectively. The first rubbery plateau follows at *E*’ values of approximately 7.4 × 10^8^ Pa, indicating sufficient chain mobility for further development crystalline regions within the PLA matrix. Subsequent increase in *E*′ corresponds to cold crystallization of PLA, while the second rubbery plateau observed above approximately 115 °C indicates increased stiffness resulting from reduction in the amorphous phase due to crystalline formation [32]. Similar observation were previously reported by Cao et al. [33] and Santonja-Blasco et al. [34].

The storage modulus curves of all PLA/PHBV blends exhibit a profile similar to that of neat PLA; however, gradual changes become evident with increasing PHBV content. All blends display characteristic regions corresponding to the glassy state, glass transition, first rubbery plateau, cold crystallization, and second rubbery plateau [32]. Comparable behavior was reported by Mofokeng and Luyt [35] for PLA/PHBV blends with different blend rations, including systems containing TiO_2_ nanofillers commonly used in biodegradable nanocomposite development [36]. Furthermore, *T*_g_ values of PLA and PHBV obtained from the *E*″ curves of the blends are generally lower than the corresponding *T*_g_ values of the neat polymers (Figure 5b). Although no pronounced composition trend *T*_g_ change was observed (Table 4), broadening of the *E*″ peaks indicated damping behavior and enhanced energy dissipation with the blend as PHBV content increases [37]. Such behavior further supports partial miscibility between PLA and PHBV and confirms mutual influence of both polymer phases on overall blend stiffness [35]. Similar peak broadening and slight shift toward *T*_g_ region of PHBV are also observed in the tan δ curves (Figure 5c), indicating enhanced damping behavior within the blends. According to Zhao et al. [38], such behavior originates from limited compatibility and partial miscibility between PLA and PHBV phases. Most blends exhibit comparable tan δ peak intensities, except PLA65/PHBV and PLA70/PHBV. Reduced peak intensity in these blends is likely associated with the higher PHBV fraction, which restricts mobility of PLA chains within the glass transition region [35]. A smaller transition observed at approximately 100 °C in the tan δ curves of neat PLA and all blends corresponds to cold crystallization of PLA. The appearance of this transition is related to increased molecular mobility during the transformation from the glassy to the rubbery state [35].

### 3.6. Mechanical Properties

**Figure 6:**
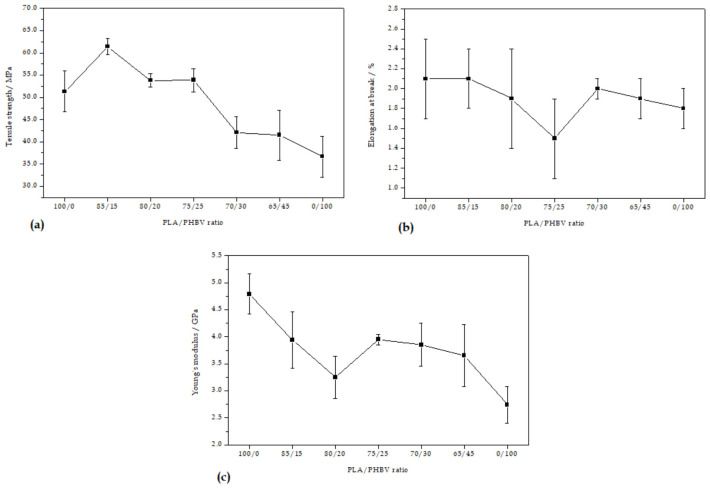
(**a**) Tensile strength; (**b**) elongation at break; (**c**) Young’s modulus values for the pure PLA and PHBV and their blends.

Mechanical properties of neat PLA, neat PHBV, and PLA/PHBV blends were evaluated by tensile testing. The obtained tensile strength, elongation at break, and Young’s modulus values are presented in Figure 6.

The tensile behavior of the investigated samples strongly depends on blend composition. Neat PLA exhibited a tensile strength of approximately 50 MPa (Figure 6a) together with the highest Young’s modulus values (Figure 6c), reflecting its relatively high stiffness. In contrast, neat PHBV showed lower tensile strength (approximately 35 MPa) and lower stiffness despite its significantly higher degree of crystallinity. The incorporation of PHBV into the PLA matrix resulted in a non-linear variation in tensile strength (Figure 6a). The PLA85/PHBV blend exhibited the highest tensile strength, reaching approximately 62 MPa. Such behavior may be attributed with improved chain packing and the nucleating effect of low PHBV concentrations within the PLA matrix. Further increase in PHBV content resulted in a gradual decrease in tensile strength, most likely due to phase separation and the increasing contribution of the brittle PHBV phase, consistent with SEM observations. Similar behavior was previously reported by Kanda et al. (2018) [39] and Gerard et al. (2014) [4]. Young’s modulus gradually decreased with increasing PHBV content, indicating that stiffness is predominantly governed by the PLA-rich phase. Such behavior is consistent with the observed morphology and partial compatibility between the components. Ma et al. (2013) [40] also observed a reduction in Young’s modulus with increasing PHBV content; however, the reported values ranged from 3.5 GPa to 0.5 GPa, which may be attributed to different polymer type and blend preparation procedures. Elongation at break values (Figure 6b) remained relatively low for all investigated samples (approximately 1.4–2.2%), confirming the brittle nature of both neat polymers and their blends. Comparable results have been reported in the literature [36,39,40], where unmodified PLA and PHBV generally exhibit elongation at break values below 10%, frequently limited to only a few percent. Limited ductility is commonly attributed with restricted chain mobility, high crystallinity of PHBV, and insufficient interfacial adhesion between the polymer phases [41]. From an application standpoint, packaging materials require not only sufficient strength but also a certain degree of flexibility to withstand stresses occurring during processing, handling, and service life. In such systems, maximizing tensile strength alone does not necessarily result in optimal overall performance. Although the PLA85/PHBV blend exhibited the highest tensile strength, relatively high stiffness and low elongation may limit practical applicability in flexible packaging systems. In contrast, the PLA65/PHBV blend exhibited a more balanced mechanical response by combining moderate strength with reduced stiffness, which may be advantageous when considering the overall functional requirements of compostable packaging materials.

### 3.7. Gas Permeability Testing

**Figure 7:**
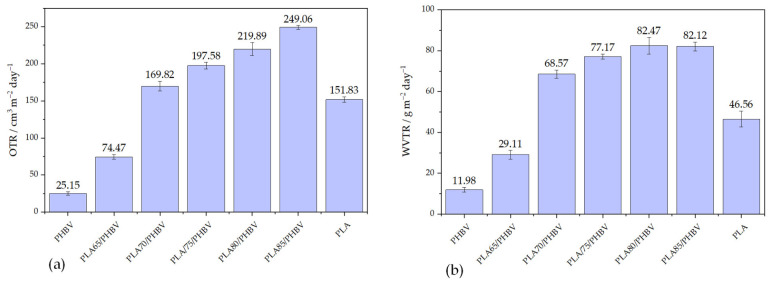
The obtained values of (**a**) OTR and (**b**) WVTR of the pure polymers and their blends.

Barrier performance of neat PLA, neat PHBV, and PLA/PHBV blends was evaluated through oxygen transmission rate (OTR) and water vapor transmission rate (WVTR) measurements, since gas barrier properties represent one of the key requirements for food packaging materials. The obtained OTR and WVTR values are presented in Figure 7.

As shown in Figure 7, neat PHBV exhibited the lowest OTR and WVTR values, amounting to 25.15 cm^3^ m^−2^ day^−1^ and 11.98 g m^−2^ day^−1^, respectively. In contrast, neat PLA showed significantly higher permeability values, with OTR and WVTR reaching 151.83 cm^3^ m^−2^ day^−1^ and 46.56 g m^−2^ day^−1^, respectively. Such behavior corresponds to approximately sixfold higher oxygen permeability and nearly fourfold higher water vapor permeability compared with PHBV.

The superior barrier performance of PHBV is primarily associated with its high degree of crystallinity (89.5%) and the presence of dense crystalline domains acting as impermeable regions within the polymer matrix. Crystalline regions increase the tortuosity of the diffusion pathway, thereby limiting penetration and transport of gas molecules through the material [42]. In contrast, the lower crystallinity of PLA (26.4%) results in a predominantly amorphous structure with increased free volume between polymer chains, facilitating diffusion of oxygen and water vapor molecules [43].

Increasing PHBV content in the blends significantly improved barrier performance, as reflected by progressive reductions in both OTR and WVTR values, which is highly relevant for food packaging applications. Although both permeability parameters generally decreased with increasing PHBV content, a slight reduction in WVTR was observed for the PLA85/PHBV blend compared with PLA80/PHBV.

Among the investigated blends, PLA65/PHBV exhibited the lowest OTR and WVTR values, reaching 74.47 cm^3^ m^−2^ day^−1^ and 29.11 g m^−2^ day^−1^, respectively. Such behavior can be partially attributed to the relatively high crystallinity of the blend. However, despite the lower crystallinity compared with neat PHBV, the improved barrier performance additionally suggests the influence of partial miscibility and favorable phase organization within the blend structure.

Although permeability generally increased with increasing PLA content, the obtained results do not show a direct linear correlation with crystallinity degree alone. Such behavior may be explained by the partial miscibility and reduced structural homogeneity of the blends. Partial phase separation, where PHBV domains are dispersed within the PLA matrix, promotes formation of interfacial voids and microcavities. Such structural discontinuities create preferential diffusion pathways that facilitate transport of oxygen and water vapor molecules through the blend structure, resulting in increased OTR and WVTR values [44].

Only a limited number of studies report detailed gas permeability behavior of PLA/PHBV blends. Perez-Martinez et al. (2024) [45] measured WVTR values for neat PHBV and a PLA/PHBV (50/50) blend and reported values of approximately 127 g m^−2^ day^−1^ and 7716 g m^−2^ day^−1^, respectively. The significant increase in permeability was attributed to poor structural homogeneity of the blend. It should be emphasized, however, that the investigated materials were prepared by solvent casting, which may considerably influence blend morphology compared with melt blending used in the present study.

Similarly, Zembouai et al. (2013) [46] observed a gradual reduction in oxygen and water vapor permeability with increasing PHBV content in PLA/PHBV blends. The incorporation of 35, 50, and 75% PHBV reduced oxygen permeability of PLA by 35.3, 43.2, and 81.5%, respectively, while water vapor permeability decreased by 22.7, 36.6, and 58.9% [46]. Jost and Kopitzky (2015) [47] also reported increasing OTR and WVTR values with decreasing PHBV content. WVTR increased from 10.1 to 14.6 g 100 μm m^−2^ day^−1^, while oxygen permeability increased from 55.3 to 79.8 cm^3^ 100 μm m^−2^ day^−1^ when PHBV content decreased from 50 to 25% [47]. This increase in permeability is explained in the mentioned research [47] by the formation of interlamellar crystallites, i.e., by the interpenetration of one phase into the other resulting in a phase-separated morphology. During the crystallization process, PHBV forms highly ordered lamellar crystals, while PLA remains predominantly amorphous, leading to limited compatibility between the two phases. As the PHBV content decreases, the continuity of the crystalline PHBV barrier phase is disrupted, and a larger interfacial area between PLA-rich and PHBV-rich domains is generated. The interpenetration of one phase into the other during crystal growth promotes the formation of irregular interlamellar regions and defective interfaces with weak interfacial adhesion. Consequently, microvoids and cavities can develop between the phases due to differences in crystallization behavior, shrinkage, and chain packing density. These interfacial defects increase the free volume within the blend and create preferential diffusion pathways for oxygen and water vapor molecules. Instead of following a highly tortuous pathway through dense crystalline regions, penetrant molecules can diffuse more easily through the amorphous regions and interfacial cavities, resulting in increased permeability. It is worth mentioning that the following research tested the permeabilities of the pure polymers as well, and PHBV showed a lot lower values than the PLA [47]. Barrier properties are particularly important for packaging of perishable food products, where the packaging material must effectively limit transport of oxygen and moisture in order to preserve product quality and stability [48]. Nevertheless, further investigation of barrier behavior in PLA/PHBV systems remains necessary in order to fully assess their applicability in food packaging applications.

Compared with other biodegradable polymers, including PBS, PBSA, and PBAT, PLA and PHBV generally exhibit superior barrier performance toward oxygen and water vapor [49]. Literature data indicate that most biodegradable polymers exhibit lower oxygen permeability than water vapor permeability, which is associated with increased moisture sorption within the polymer matrix and may simultaneously facilitate biodegradation processes [49]. Conventional petroleum-based packaging polymers such as low-density polyethylene (LDPE), high-density polyethylene (HDPE), and polypropylene (PP) generally exhibit higher gas permeability compared with biopolymers. Nevertheless, extensive industrial application of such materials is largely associated with low production cost and favorable mechanical performance, while gas barrier properties are commonly enhanced through multilayer structures containing aluminum barrier layers [49].

### 3.8. Biodegradable Properties

Assessment of biodegradation behavior is essential for the development of sustainable materials with reduced environmental impact. Biodegradation is a microbially driven process in which polymeric materials are converted into smaller molecules and ultimately mineralized into carbon dioxide, water, and other low-molecular-weight products [50,51,52,53]. The process involves microbial colonization of the polymer surface, enzymatic depolymerization, assimilation of degradation products, and subsequent mineralization.

The rate and mechanism of biodegradation strongly depend on environmental conditions, including temperature, pH, moisture content, and oxygen availability, as well as on polymer structure and morphology [50,53]. Although both PLA and PHBV are classified as biodegradable polymers, they exhibit significantly different degradation behaviors. PHBV generally undergoes faster biodegradation due to its higher susceptibility to enzymatic hydrolysis and microbial attack, whereas PLA degrades considerably more slowly under similar environmental conditions [54,55]. In marine environments, PHBV may degrade within weeks to months, while PLA often exhibits negligible degradation over prolonged periods [54,55]. Biodegradation of PLA is strongly temperature-dependent and becomes significantly accelerated under industrial composting conditions at approximately 60 °C, whereas degradation at lower temperatures remains limited [56].

**Figure 8:**
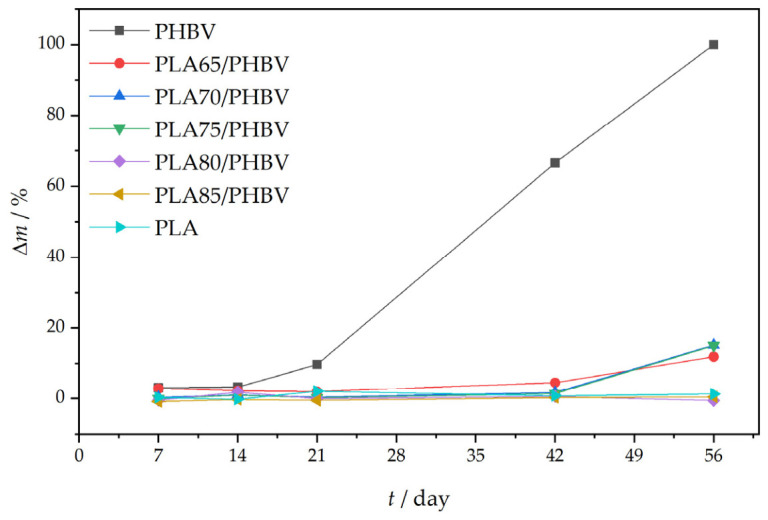
Mass change rates of the pure PHBV, pure PLA, and PLA/PHBV blends during the biodegradation process.

Figure 8 presents mass loss values of neat PHBV, neat PLA, and PLA/PHBV blends during 56 days of biodegradation in compost soil. Among all investigated materials, neat PHBV exhibited the highest biodegradation rate, reaching complete degradation after 56 days, corresponding to 100% mass loss (Figure 8, black curve). Although literature reports indicate substantial biodegradation of PHBV under composting conditions, complete degradation within such a relatively short period has rarely been reported. Deronié et al. [57] observed approximately 36% mass loss after 180 days for PHBV samples with 8 wt.% hydroxyvalerate content and a thickness of 200 μm, while Zaidi et al. [58] reported only 0.5% mass loss after 112 days for 1 mm thick PHBV films containing 3 mol% hydroxyvalerate. Similarly, Reay et al. [59] reported PHBV degradation in soil ranging between 1.5 and 5%.

The significantly enhanced degradation observed in the present study may be attributed to the highly biologically active compost environment. The compost soil exhibited favorable pH and moisture conditions, while the intentionally introduced microbial strains, together with indigenous soil microorganisms, formed a diverse microbial consortium capable of promoting efficient enzymatic degradation of PHBV. In addition, the relatively low film thickness used in the present study likely contributed to accelerated biodegradation by increasing surface area available for microbial colonization, moisture penetration, and biofilm formation [60].

In contrast to PHBV, neat PLA exhibited very limited biodegradation (Figure 8, light-blue curve), with only 1.29% mass loss after 56 days. Such behavior confirms the considerably slower degradation kinetics of PLA under moderate composting conditions. Brunšek et al. [61] reported approximately 3.99% mass loss of PLA after 11 days under controlled composting conditions at 29 ± 1 °C and soil moisture content of 60 ± 5%, indicating that even relatively small variations in environmental conditions can strongly influence PLA biodegradation behavior. The low degradation rate of PLA is primarily associated with its relatively high crystallinity, which limits water penetration and reduces accessibility of polymer chains to microbial and enzymatic attack [61].

For PLA/PHBV blends, increasing PHBV content resulted in progressively higher mass loss values after 56 days of biodegradation. Blends containing higher PHBV fractions (PLA65/PHBV, PLA70/PHBV, and PLA75/PHBV) exhibited mass losses ranging from 11.91 to 15.21%, whereas PLA-rich blends (PLA80/PHBV and PLA85/PHBV) showed negligible or slightly negative mass changes, most likely caused by residual soil particles or moisture accumulation within surface defects.

The obtained results indicate that biodegradation within the blends proceeds preferentially through the PHBV phase, which is considerably more susceptible to microbial degradation. As degradation progresses, removal of PHBV domains generates pores and diffusion pathways within the PLA matrix, facilitating additional structural deterioration. Similar behavior has previously been reported for PLA/PHBV systems, where PHBV acts as the biodegradable phase dispersed within the more degradation-resistant PLA matrix [62,63,64].

**Figure 9:**
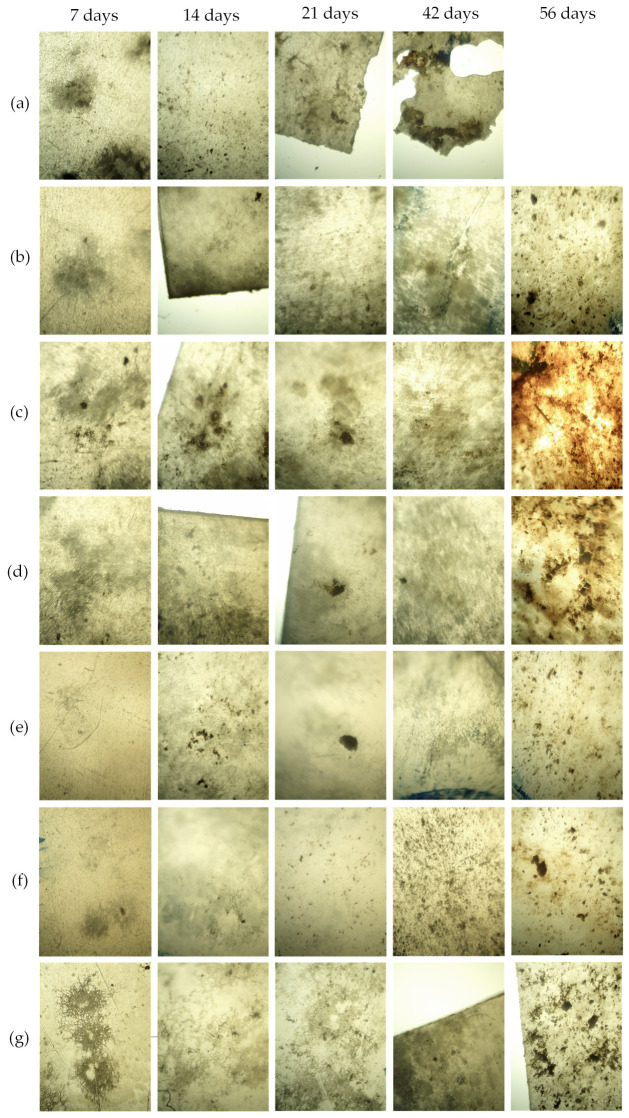
Microphotographs (optical microscope) of (**a**) pure PHBV; (**b**) PLA65/PHBV; (**c**) PLA70/PHBV; (**d**) PLA75/PHBV; (**e**) PLA80/PHBV; (**f**) PLA85/PHBV; and (**g**) pure PLA after 7, 14, 21, 46, and 56 days of the biodegradation process.

**Figure 10:**
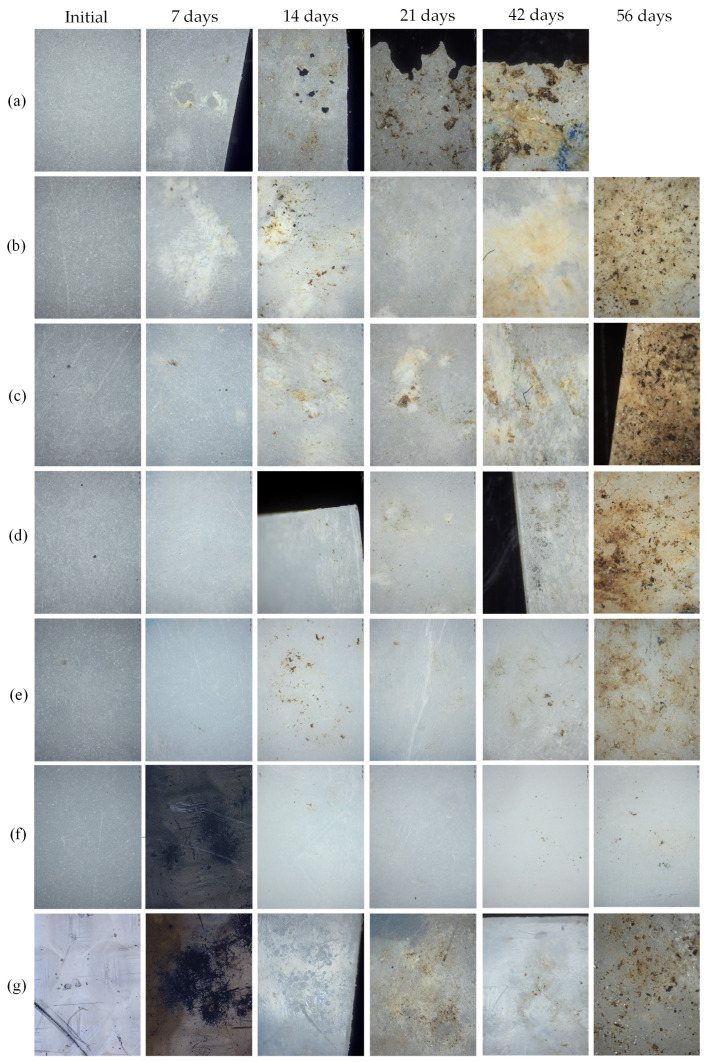
Microphotographs (polarizing microscope) of (**a**) pure PHBV; (**b**) PLA65/PHBV; (**c**) PLA70/PHBV; (**d**) PLA75/PHBV; (**e**) PLA80/PHBV; (**f**) PLA85/PHBV; and (**g**) pure PLA after 7, 14, 21, 46, and 56 days of the biodegradation process.

Morphological changes occurring during biodegradation were further analyzed through optical and polarized light microscopy, and the obtained micrographs are presented in Figure 9 and Figure 10. Progressive microbial colonization of the sample surfaces becomes increasingly evident with biodegradation time. Micrographs recorded after 56 days exhibit the highest density of brown-colored colonies, which may correspond to microbial growth and residual soil particles trapped within surface defects and cracks. Filamentous gray structures observed in Figure 9 additionally indicate fungal growth on the polymer surfaces.

Microbial colonization was observed for all investigated materials; however, considerably fewer colonies were detected on PLA80/PHBV (Figure 9e) and PLA85/PHBV (Figure 9f), consistent with their limited biodegradation behavior. Such observations confirm the initiation of biodegradation processes in all investigated systems, although degradation rates differed substantially depending on blend composition [63].

Only neat PHBV exhibited pronounced structural deterioration during the biodegradation process, visible through fragmentation and material loss along the sample edges after 21 and 42 days. After 56 days, no PHBV sample remained for microscopic observation due to complete degradation, which is consistent with the measured 100% mass loss. At the beginning of the experiment, all sample surfaces appeared relatively smooth; however, progressive surface erosion and increased roughness were observed during biodegradation for most compositions except PLA85/PHBV (Figure 10). Such surface deterioration is commonly associated with progressing biodegradation [65].

Only minor morphological changes were observed for PLA80/PHBV (Figure 10e) and neat PLA after 42 and 56 days, corresponding to their low measured mass loss values. Unlike neat PHBV, no visible fragmentation was detected for the blends or PLA samples, further confirming the considerably slower biodegradation kinetics of PLA-rich systems, consistent with previously reported studies [6,12,13].

**Figure 11:**
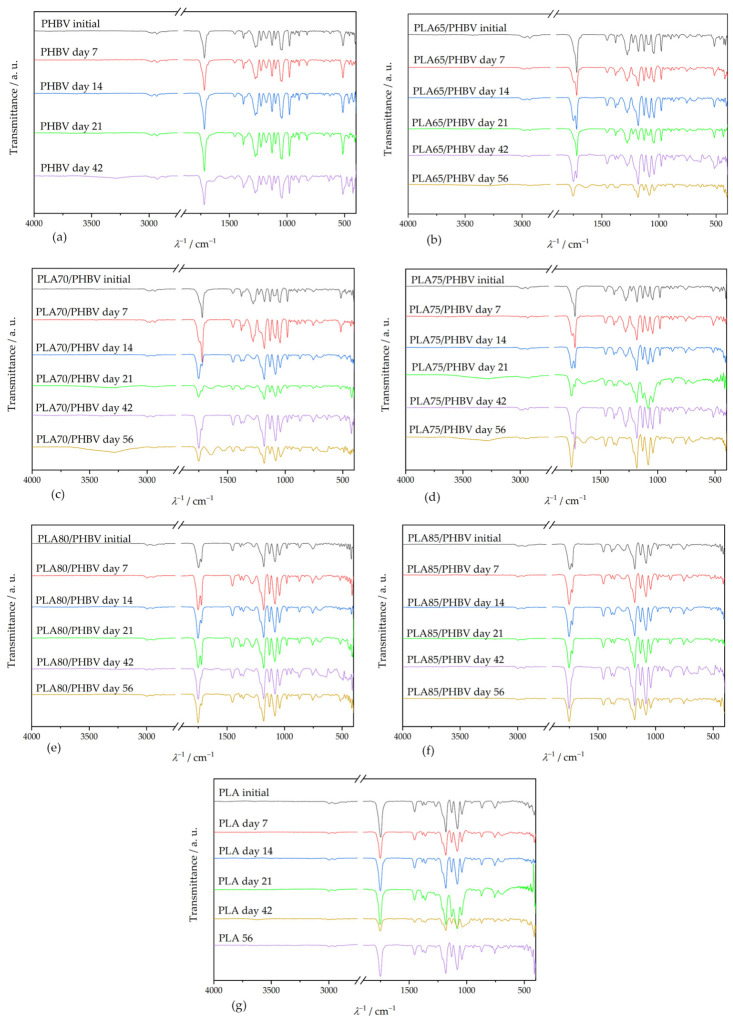
FTIR spectra obtained before (initial) and after 7, 14, 21, 42, and 56 days of biodegradation of (**a**) the pure PHBV; (**b**) PLA65/PHBV; (**c**) PLA70/PHBV; (**d**) PLA75/PHBV; (**e**) PLA80/PHBV; (**f**) PLA85/PHBV; and (**g**) the pure PLA.

To further confirm biodegradation processes, ATR-FTIR spectra of all samples were recorded after 7, 14, 21, 42, and 56 days of compost exposure (Figure 11). Several characteristic absorption bands of PHBV (Figure 11a) and PLA (Figure 11g) are also visible in spectra of the corresponding blends. Absorption bands in the region between 3000 and 2850 cm^−1^ correspond to C–H stretching vibrations, while bands between 1450 and 1350 cm^−1^ are associated with C–H bending vibrations. The spectral region between approximately 950 and 1200 cm^−1^ corresponds to C–O stretching vibrations characteristic of both polymers. The main distinction between PLA and PHBV spectra is observed in the carbonyl absorption bands located at approximately 1720 cm^−1^ for PHBV and 1750 cm^−1^ for PLA [21,22].

Progress of biodegradation is primarily confirmed through shifts in the carbonyl absorption bands toward higher wavenumbers, accompanied by reduced peak intensity and increased peak sharpness [65]. Such spectral changes are visible for both neat PHBV and neat PLA; however, they are considerably more pronounced for PHBV, confirming its significantly higher biodegradation susceptibility.

ATR-FTIR spectra of PLA/PHBV blends contain two characteristic carbonyl absorption bands corresponding to PLA and PHBV phases. The initial spectrum of PLA65/PHBV (Figure 11b) exhibits a dominant carbonyl peak associated with PHBV. During biodegradation, gradual reduction in intensity of the PHBV carbonyl peak occurs simultaneously with increased intensity of the PLA carbonyl peak, indicating preferential degradation of the PHBV phase and increasing dominance of the remaining PLA structure [56].

Similar spectral behavior was observed for PLA70/PHBV (Figure 11c) and PLA75/PHBV (Figure 11d), supporting the measured mass loss values of approximately 15% resulting from degradation of the PHBV-rich phase. Broad absorption bands observed between approximately 3200 and 3500 cm^−1^ correspond to O–H stretching vibrations originating from adsorbed water molecules.

In contrast, ATR-FTIR spectra of PLA80/PHBV (Figure 11e) and PLA85/PHBV (Figure 11f) exhibit only minor reductions in PHBV carbonyl peak intensity without noticeable shifts toward higher wavenumbers. Such behavior further confirms the limited biodegradation observed for PLA-rich blends, consistent with the negligible mass loss values measured during compost exposure.

## 4. Conclusions

The results of this study demonstrate that PLA/PHBV blends exhibit composition-dependent structure–property–performance relationships relevant to compostable packaging applications. The SEM analysis revealed relatively uniform morphologies without pronounced large-scale phase separation, indicating a certain degree of homogeneity and partial interfacial adhesion between PLA and PHBV. FTIR-ATR analysis further supported interactions between the two polyesters through changes in characteristic absorption bands. Thermal analysis confirmed the semicrystalline nature of both polymers, with PHBV exhibiting a higher degree of crystallinity. The presence of PHBV promoted PLA crystallization, indicating its nucleating effect within the blends. In contrast, thermogravimetric analysis showed that PLA contributes to improved thermal stability, with higher PLA content (≥80 wt.%) enhancing resistance to thermal degradation. Barrier performance was strongly composition-dependent, with increasing PHBV content leading to lower OTR and WVTR values. The PLA65/PHBV blend exhibited the most favorable barrier properties, which can be attributed to its balanced phase structure and relatively high crystallinity. This composition also showed the highest biodegradation rate, primarily due to the presence of the highly biodegradable PHBV phase. Mechanical testing indicated that PLA-rich blends provide higher strength and stiffness, while increasing PHBV content contributes to enhanced flexibility. The observed biodegradation behavior follows the compositional distribution of the polymer phases, with degradation proceeding preferentially through the PHBV-rich domains. Considering the requirements of food packaging materials, including mechanical integrity, barrier performance, and stability during use, further efforts should be directed toward optimizing phase compatibility and interfacial interactions within the blends. Achieving improved structural homogeneity is essential for balancing functional performance with controlled biodegradation, thereby supporting the development of reliable compostable packaging materials for food applications.

## Figures and Tables

**Table 1:** Specifications of PLA and PHBV.

Property	PLA	PHBV
Melt flow index (g (10 min)^−1^)	3–6	5–15
Tensile strength at break (MPa)	45	39
Elongation at break (%)	≤5	2
Young’s modulus (MPa)	3500	2800–3500
Melting temperature (°C)	155	170–176

**Table 2:** Results of DSC analysis of the tested samples.

Sample	*T*_g,PHBV_/°C	*T*_g,PLA_/°C	*T*_cc,PHBV_/°C	Δ*H*_cc,PHBV_/J g^−1^	*T*_cc,PLA_/°C	Δ*H*_cc,PLA_/J g^−1^	*T*_m,PLA_/°C	Δ*H*_m,PLA_/J g^−1^	*T*_m,PHBV_/°C	Δ*H*_m,PHBV_/J g^−1^	*T*_c_/°C	Δ*H*_c_/J g^−1^	*χ*_c,PLA_/%	*χ*_c,PHBV_/%
PHBV	-	-	-	-	-	-	-	-	171.9	97.6	125.1	88.9	-	89.5
PLA65/PHBV	0.9	58.3	41.5	2.0	-	-	152.7	3.8	170.9	22.1	118.1	15.0	6.3	60.0
PLA70/PHBV	1.5	59.1	42.0	4.7	123.5	9.0	153.9	1.9	172.6	16.7	-	-	2.9	21.9
PLA75/PHBV	1.4	58.0	40.5	2.2	125.4	11.7	153.7	3.2	171.3	11.3	-	-	4.6	13.8
PLA80/PHBV	1.2	58.9	37.3	0.4	126.1	10.8	153.4	5.3	171.0	7.5	-	-	7.1	34.6
PLA85/PHBV	1.4	58.6	-	-	128.1	9.4	154.0	6.5	171.7	3.2	-	-	8.2	19.6
PLA	-	59.5	-	-	116.9	28.7	151.4	24.6	-	-	-	-	26.4	-

**Table 3:** Results of TG analysis of the tested samples.

Sample	TG					DTG	
	*T*_onset_/°C	*T*_end_/°C	Δ*m*(PHBV)/%	Δ*m*(PLA)/%	*R*_700°C_/%	*T*_max_(PHBV)/°C	*T*_max_(PLA)/°C
PHBV	282.4	303.0	98.7	-	1.3	298.0	-
PLA65/PHBV	283.1	378.1	36.5	62.5	0.7	295.9	367.8
PLA70/PHBV	243.3	336.0	34.5	64.0	0.6	299.8	371.1
PLA75/PHBV	261.8	306.5	25.8	69.3	4.4	292.6	332.5
PLA80/PHBV	288.0	380.5	21.2	75.3	0.4	300.4	369.7
PLA85/PHBV	285.9	380.0	16.4	79.8	1.3	299.7	369.1
PLA	348.5	377.1	-	99.2	0.4	-	370.4

**Table 4:** Glass transition temperatures (*T*_g_) of PLA and PHBV obtained from tanδ curve and *E*″ curve for the pure PLA and PHBV, and their blends.

	*T*_g_ (PHBV, tanδ Curve)/°C	*T*_g_ (PLA, tanδ Curve)/°C	*T*_g_ (PHBV, *E*″ Curve)/°C	*T*_g_ (PLA, *E*″ Curve)/°C
PHBV	14.3	-	10.4	-
PLA65/PHBV	-	66.9	−0.7	60.9
PLA70/PHBV	-	66.9	−1.1	60.4
PLA75/PHBV	-	66.8	−1.2	60.3
PLA80/PHBV	-	67.2	−3.1	60.7
PLA85/PHBV	-	67.1	-	61.1
PLA	-	68	-	62.4

## Data Availability

The raw data supporting the conclusions of this article will be made available by the authors on request.
